# Silicon uptake via the transporters SySIT-L and SyLsi-L enhances the growth and photosynthesis of *Synechococcus* sp. PCC 7002

**DOI:** 10.1128/mbio.01844-25

**Published:** 2025-08-25

**Authors:** Daixi Liu, Bokun Chen, Yue Meng, Yafei Wang, Wei Zhao, Hongli Ji, Xue Yang, Minghao Zhu, Liwen Zheng, Gang Li, Jihua Liu

**Affiliations:** 1 State Key Laboratory of Discovery and Utilization of Functional Components in Traditional Chinese Medicine, Key Laboratory of Chemical Biology (Ministry of Education), Shandong Basic Science Research Center (Pharmacy), School of Pharmaceutical Sciences, Cheeloo College of Medicine, Shandong University 12589, Jinan, China; 2 Institute of Marine Science and Technology, Shandong University 520252, Qingdao, China; 3 Qingdao Key Laboratory of Ocean Carbon Sequestration and Negative Emission Technology, Shandong University, Qingdao, China; 4 Daya Bay Marine Biology Research Station, South China Sea Institute of Oceanology, Chinese Academy of Sciences 74718, Guangzhou, China; 5 Global Ocean Negative Carbon Emissions (ONCE) Program Alliance, Qingdao, China; McMaster University, Hamilton, Ontario, Canada

**Keywords:** *Synechococcus*, Si accumulation, Si transporter, cell growth, photosynthetic physiology

## Abstract

*Synechococcus,* a type of picoplankton, plays a crucial role in the carbon (C) and silicon (Si) biogeochemical cycles of the ocean. Their contribution to biological Si within the oligotrophic oceans can be comparable to that of diatoms. However, the mechanisms of Si assimilation, accumulation, and its impact on cellular metabolism in *Synechococcus* remain poorly understood. Here, we analyzed the physiological and transcriptomic responses of a model strain *Synechococcus* sp. PCC 7002 in the exponential growth phase to gradient Si enrichment conditions (0, 25, 50, 120, and 200 μmol L^−1^) and performed knockouts of Si transport genes *SySIT-L* and *SyLsi-L* to assess relevant function. Results showed that the specific growth rate over 5 days of cultivation was increased by up to 37% in response to Si enrichment under the concentration of 120 μM, accompanied by the physiological parameters, such as cellular content of biological Si and chlorophyll *a*, as well as elevated rates of photosynthetic O_2_ evolution and dark respiration, both of which increased with increasing ambient Si concentration especially on day 1. These changes were corroborated by the transcriptomic analysis. Knockout of the *SySIT-L* and *SyLsi-L* genes reduced the cellular Si content by ~80% both on days 1 and 5. Additionally, we found that two Si transporters were widespread in 469 sequenced cyanobacterial genomes. This study provides new scientific evidence from physiological and metabolic perspectives on the role of *Synechococcus* in the marine Si and C cycles, serving as a valuable starting point for exploring the mechanisms of Si metabolism in picoplankton.

**IMPORTANCE:**

This work first reveals the silicon uptake in *Synechococcus* PCC 7002 via two silicon transporters SIT-L and Lsi-L, which are widely distributed in 469 sequenced cyanobacterial genomes. This enhances photosynthesis and respiration, thus promoting cell growth. Our study serves as a valuable starting point for exploring the mechanisms of silicon metabolism in *Synechococcus*, providing biological evidence to explain the silicon accumulation of cyanobacteria in the oceans.

## INTRODUCTION

The silicon (Si) cycle plays a regulated role in the global atmospheric carbon dioxide concentration over long time scales (1), being closely related to the carbon cycle, the biological pump, and the circulation of various metallic elements (e.g., iron, aluminum, magnesium, and germanium) in the ocean. It has received widespread attention in recent decades (2, 3). *Synechococcus*, as an important picophytoplankton, is widely distributed in the global oceans from the equator to the cold zone (4, 5), especially in the oligotrophic oceans where the cell abundance is up to 10^9^ cells L^−1^. Recent studies have shown that *Synechococcus* also plays a key role in the marine Si cycle in some areas, such as the equatorial Pacific Ocean and the Sargasso Sea, contributing 20% of the total biological silicon (bSi) within the euphotic layer. Its contribution is even comparable to diatoms (6, 7). This underscores the need to reevaluate the contribution of *Synechococcus* to the biological carbon pump and nutrient cycling, as the uptake and accumulation of Si by cells may affect growth, metabolism, and cell sinking, regulating both photosynthetic carbon fixation and the biological carbon pump (8, 9).

Si accumulation in *Synechococcus* was first reported by Baines et al. (6), and then a series of relevant studies on the contribution of marine picoplankton to bSi standing stocks and production were carried out successively. In the North Atlantic, the cellular silicon content of *Synechococcus* at different depths varied by thousands of times and had no significant relationship with ambient Si concentration. This variation might be related to differences in community structure (10). In the Sargasso Sea, the Si production rate of picoplankton accounted for 9% of the total, with 55% of this contribution attributed to *Synechococcus* (11). In the Southern Equatorial Pacific, picoplankton, with *Synechococcus* as the dominant group, account for approximately 11%–26% of the total bSi stock (12). Laboratory simulations revealed that the cellular Si content of six species belonging to *Synechococcus* spp. was increased with increasing ambient Si concentration (0–120 µM), but Si enrichment showed no significant effect on cell growth rate (13). Regrettably, the mechanism by which exogenous Si enters the *Synechococcus* cell and affects cellular metabolism is still unknown.

In eukaryotes, the SIT family of Si transporters was initially identified in diatoms, located in the plasma membrane and playing a key role in cellular silicon absorption (14). Diatoms harbor a variety of SITs, each exhibiting distinct gene expression profiles, suggesting their potential involvement in diverse aspects of the silicification process (15). For higher plants, two types of Si transporters have been identified: channel-type silicon transporters (Lsi1 type) and efflux silicon transporters (Lsi2 type) (16, 17). Lsi1 is a member of the Nod26-like intrinsic protein subfamily within the aquaporin family, while Lsi2 is a predicted anion transporter. Lsi1 is located on the distal side of the exodermis and endodermis, mediating Si influx into root cells, whereas Lsi2 is localized on the proximal side, facilitating Si efflux to the apoplast. The coordinated action of Lsi1 and Lsi2 is essential for efficient silicon uptake in rice. Besides eukaryotes, sequences with similarity to SITs were also found in *Synechococcus* (18). However, the function of these SIT-like (SIT-L) proteins has not been verified or explored.

*Synechococcus* sp. PCC 7002 belongs to the 5.1 clade of *Cyanobacteria* and is widely distributed in various aquatic environments, including oceans, freshwater lakes, and rivers, especially in nearshore regions with higher Si concentration. In this study, *Synechococcus* sp. PCC 7002 was cultured under five dissolved Si levels (0–200 µM) at suitable light and temperature conditions to evaluate changes in cell composition, photosynthesis, and gene expression through transcriptomic analysis affected by Si enrichment. Additionally, we performed gene knockout on two Si transporters (SySIT-L and SyLsi-L) to explore the mechanism underlying Si uptake. This research provides insights into the impact of Si uptake on physiological processes in *Synechococcus*. Furthermore, it contributes a novel perspective on the role of picoplankton in the marine Si cycle.

## MATERIALS AND METHODS

### Culture protocol

The cyanobacteria *Synechococcus* sp. strain PCC 7002, originally obtained from the Pasteur Culture Collection (Paris, France), was cultivated using an improved formulation of A+ medium (19) in 75 cm^2^ disposable cell culture bottles under the following conditions: 30°C, 120 rpm, 12:12 light:dark cycle, and 150 µmol photons m^−2^ s^−1^ light intensity. The exact composition of A+ medium is 45 g L^−1^ NaCl, 1.5 g L^−1^ KCl, 2.5 g L^−1^ NaNO_3_, 12.5 g L^−1^ MgSO_4_·7H_2_O, 0.695 g L^−1^ CaCl_2_, 136 g L^−1^ KH_2_PO_4_, 0.239 g L^−1^ NaVO_3_, 121.1 g L^−1^ Tris/HCl (pH 8.2), 100 mg L^−1^ vitamin B12, 15 mM Fe-EDTA, and 1 mL L^−1^ D6 trace metal solution. D6 trace metal solution (1,000×) contains the following substances: 2.86 g L^−1^ H_3_BO_3_, 1.81 g L^−1^ MnCl_2_·4H_2_O, 0.222 g L^−1^ ZnSO_4_·7H_2_O, 1.26 g L^−1^ NaMoO_4_·2H_2_O, 0.079 g L^−1^ CuSO_4_·5H_2_O, and 0.0403 g L^−1^ CoCl_2_·6H_2_O. The source of Si, a pre-prepared 0.25 M Na_2_SiO_3_·9H_2_O stock solution, was added to the *Synechococcus* sp. PCC 7002 culture during the exponential growth phase with five final concentration gradients, i.e., 0 µmol L^−1^ (Si—0 μM), 25 µmol L^−1^ (Si—25 μM), 50 µmol L^−1^ (Si—50 μM), 120 µmol L^−1^ (Si—120 μM), and 200 µmol L^−1^ (Si—200 μM), each with three replicates per treatment. We did not handle any glass containers containing Si during the experiment to avoid contamination. We used plastic culture flasks and cleaned all equipment and materials with ultrapure water. Sampling was conducted daily at 10:00 a.m. to determine the growth rate since the initiation of Si treatment, with additional sampling performed on both days 1 and 5 to assess physiological responses.

### Growth rate

To ensure acclimation to experimental conditions, all strains were grown under the respective Si concentrations for more than 10 generations prior to physiological measurements. The inoculation concentration for each treatment was standardized at 1 × 10^6^ cells/mL. At the end of each growth cycle, the cell densities were about 2 × 10^7^ cells/mL. Then, the above-acclimated strains were used as seeds for the subsequent Si-addition experiments. After Si addition, 1 mL duplicate cultures were taken from each bottle each morning (10:00 a.m., 2 h after light on) and fixed with glutaraldehyde at a final concentration of 1% to measure the cell abundance by an Accuri C6 flow cytometer (Accuri C6, Becton-Dickinson, USA). The specific growth rate (*μ*) based on cell counting was calculated as


μ=[ln(N1)−ln(N0)]/(t1−t0),

where *N*1 denotes the cell abundance value on day 5, and *N*0 denotes the cell abundance value on day 1, respectively.

### Cell compositions

To measure the cellular Si content, 10 mL cultures were prepared in an enzyme-free centrifuge tube at 4°C and 12,000 rpm for 35 min and then washed with clean A+ medium three times to remove surface-bound Si ions. After the washes, we resuspended the cells in 15 mL of extraction buffer (20 mM Tris-HCl, pH 8.0) and applied three rounds of sonication. Each sonication cycle lasted for 1 min at 70% amplitude and repeated after a 1-min interval. The cytoplasmic soluble components in the supernatant were obtained by filtration through a 0.2 µm phosphatidylcholine (PC) membrane (25 mm in diameter) after centrifugation for 30 min at 4°C, and the Si content was measured by a segmented flow analyzer (AA3 HR Autoanalyzer, SEAL Analysis Ltd., China).

To measure cellular chlorophyll *a* content, 2 mL of culture was vacuum-filtered onto a 0.2 µm PC membrane and extracted in the dark for 12 h with 4 mL of MgCO_3_-saturated 90% acetone (vol/vol) at 4°C. After centrifugation at 4°C for 10 min (4,000 rpm), the optical absorbance of the supernatant was measured spectrophotometrically at 630, 664, and 750 nm. The content of Chl *a* was calculated as


$$Chl\;a=11.47\times (A_{664}-A_{750})-0.4\times (A_{630}-A_{750}).$$

### Physiological measurements

To measure chlorophyll fluorescence and photosynthetic O_2_ evolution rate, 10 mL of culture was taken from each bottle, dark-acclimated at 30°C for 15 min, and then measured in 3 mL to obtain the rapid light curve (RLC) of photochemical quantum yield and the photosynthetic O_2_ evolution rate, respectively. Chlorophyll fluorescence was measured by a fluorometer (FastOcean Act 2, Chelsea Technologies, UK). The maximal photochemical quantum yield of photosystem II (PS II) (*F*_*V*_/*F*_*M*_) was determined by measuring the maximum fluorescence (*F*_*M*_) and minimum fluorescence (*F*_*O*_) of the dark-acclimated cells using a saturating blue light pulse (3,000 µmol photons m^−2^ s^−1^, 1 s) in the presence of a weak modulated measuring light. The *F*_*V*_/*F*_*M*_ was calculated as


$$F_{V}/F_{M}=(F_{M}-F_{O})/F_{M},$$

Net photosynthetic O_2_ evolution rate (*P*_net_) and dark respiration rate (*R*_dark_) were obtained by a liquid O_2_ electrode (Chlorolab 2^+^, Hanshatech, USA) at the photosynthetically active radiation (PAR) of 150 and 0 µmol photons m^−2^ s^−1^, respectively.

### Transcriptome and qPCR

To evaluate the effects of Si enrichment on metabolic regulation on day 5, 15 mL culture taken from each bottle was vacuum-filtrated onto a 0.2 µm PC membrane, and the total RNA was extracted by a Bacteria RNA Extraction Kit (Vazyme, China) for the transcriptome analysis and quantitative reverse transcription-polymerase chain reaction (qPCR) validation. A transcriptomic library of 15 samples was sequenced using the Illumina HiSeq platform and quality controlled using the Trimmomatic software. Gene function was annotated according to the full genome of PCC 7002 in National Center for Biotechnology Information (NCBI) database. Differential expression analysis of genes was performed using the DEG seq package 2 (20). To ensure rigorous identification, we set thresholds of adjusted *P*-value < 0.05 and log_2_ (fold change) > 1 or log_2_ (fold change) < −1. The physiological function of differently expressed genes was analyzed using the Kyoto Encyclopedia of Genes and Genomes (KEGG) database.

In qPCR validation, cDNA was synthesized using HiScript III RT SuperMix (+gDNA wiper, Vazyme, China), and then qPCR reactions were performed using ChamQ Universal SYBR qPCR Master Mix (Vazyme, China). The primers of target genes and reference gene *rnpA* (*SYNPCC 7002_A0989*, encodes the RNase P protein subunit) are listed in Table S1. The Light Cycler 480 II sequence detection system (Roche, China) was used for conducting the reactions. We analyzed the results using the 2^-∆∆CT^ method (21).

### Identification of a putative Si transporter in PCC 7002

The putative Si transporter in *Synechococcus* sp. PCC 7002 was identified using hmmsearch. Previously reported Si transporters were used to prepare the HMM model based on known protein domains. Then, hmmsearch was executed to align the HMM model with the genome sequence database of *Synechococcus* sp. PCC 7002, setting an *E*-value threshold of 1e-5. The selected sequences were further validated by phylogenetic analysis and conserved motifs search. The phylogenetic tree was constructed by MEGA 11.0, and the conserved motifs were searched by Multiple Em for Motif Elicitation. Finally, the putative Si transporters in *Synechococcus* sp. PCC 7002 were aligned by Clustal Omega.

### Strain construction

The Δ*SySIT-L* and Δ*SyLsi-L* mutants of *Synechococcus* sp. PCC 7002 were constructed by homologous recombination. Taking Δ*SySIT-L* as an example, two gene segments immediately upstream and downstream of the *SySIT-L* gene, about 1,000 bp long, were amplified by PCR from the genomic DNA of the wild-type strain with the primer sets *SySIT-L-del-1/SySIT-L-del-2* and *SySIT-L-del-3/SySIT-L-del-4* (Table S1). A kanamycin resistance cartridge was excised from the plasmid pET30a with the primers *Kan-F/Kan-R*. All the above three fragments were joined via fusion PCR, with the kanamycin resistance cartridge in the middle. Then, the fusion fragment was cloned into the pJET1.2 blunt vector (Thermo, Beijing, China). The constructed knockout plasmid was introduced into the wild-type strain via natural transformation. First, cells were pre-cultivated at 30°C under the irradiance of 150 µmol photons m^−2^ s^−1^ for 24 h. Then, the culture was transferred to an A^+^ plate with 50 µg/mL kanamycin and incubated for a week until single colonies formed. Finally, the obtained mutants were verified through PCR and sequencing. The primers used are listed in Table S1. The recombinant plasmid was verified, and the mutants were screened to ensure the accuracy of the gene knockout. The Δ*SyLsi-L* mutant was constructed similarly. The complemented strains were constructed by inserting the *SySIT-L* and *SyLsi-L* genes into the NS1 site (CyanoBase: SYNPCC7002_A0933) of the knockout strain.

### Statistical analysis

The parameters of the photosynthetic rate versus irradiance (*P* versus I) curves were obtained using the model and by fitting the data iteratively:


$$P=I/(a\times I^{2}+b\times I+c),$$

where *P* is the relative electron transport rate (rETR) or gross photosynthetic rate (*P*_gross_), *I* is the PAR (μmol photons m^−2^ s^−1^), and *a*, *b*, and *c* are the adjustment parameters. The initial slope (*α*), the maximum production rate (rETR_max_ for chlorophyll fluorescence and *P*_max_ for photosynthetic O_2_ evolution rate), and the light saturation parameters (*E*_*K*_) are expressed as a function of *a*, *b*, and *c* parameters as follows:


$$\alpha =1/c;rETR_{max}\;or\;P_{max}=1/[b+2(a\times c{)}^{1/2}];E_{k}=(c/a{)}^{1/2},$$

We also calculated the carbon use efficiency (CUE), which represents the fraction of fixed carbon that is available for allocation to growth and can be estimated from the rates of photosynthesis and respiration according to reference 22:


$$CUE=1-[R_{dark}/(R_{dark}+P_{net})],$$

Data were presented as means and standard deviations (mean ± SD) in figures and tables. The distribution of Si transporter genes in cyanobacterial genomes was analyzed using the Cytoscape platform (23), and the diagram of metabolic pathways was created using Illustrator CC mapping software (Adobe). The *t*-test, one-way analysis of variance (ANOVA) with Tukey post-tests (Prism 5, GraphPad software), and linear fit comparisons were used to detect significant differences between different treatments. The confidence level for the statistical test was set at 0.05.

## RESULTS

### Physiological response of *Synechococcus* sp. PCC 7002 to Si enrichment

**Fig 1:**
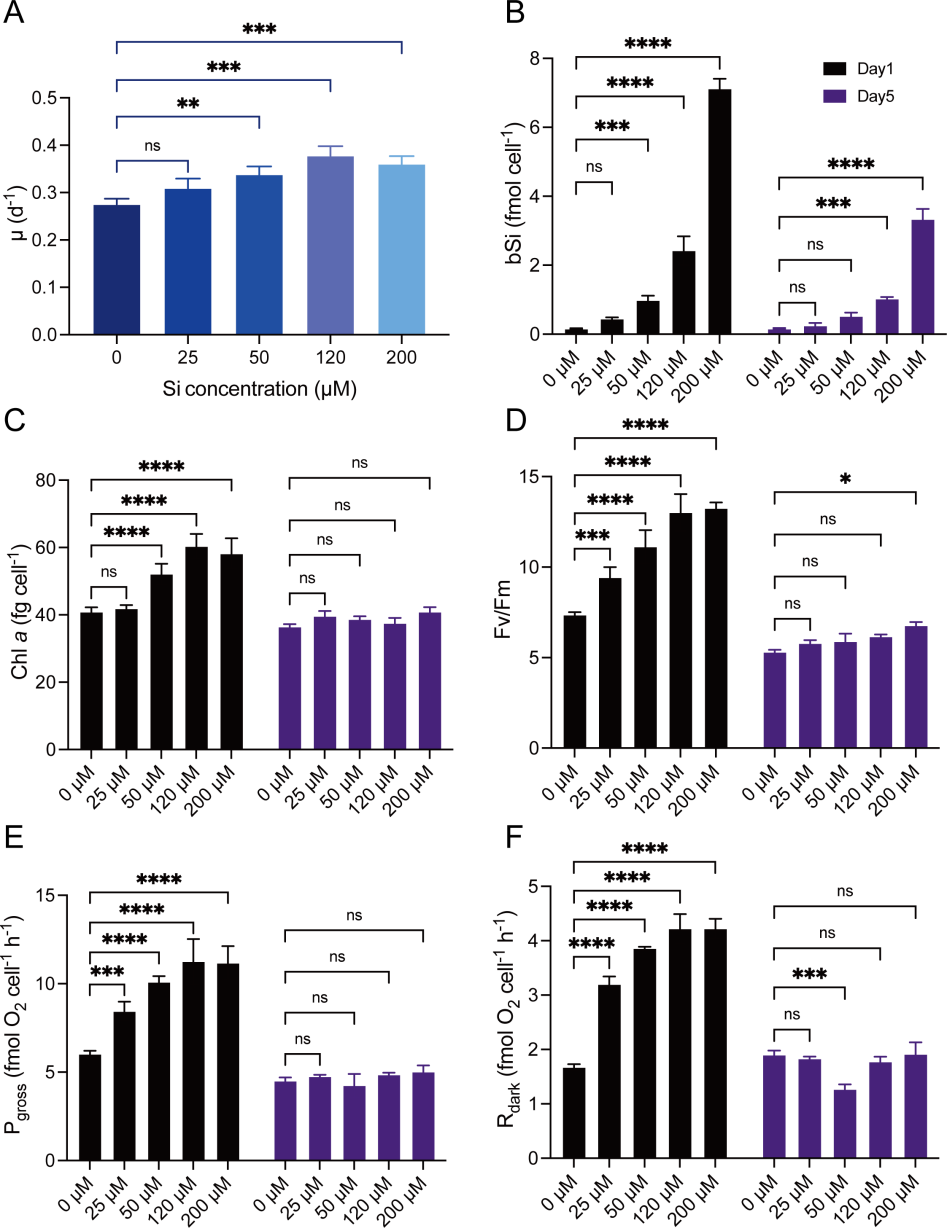
Physiological changes in *Synechococcus* sp. PCC 7002 grown under 0–200 µM silicon enrichment on days 1 and 5. (**A**) Specific growth rate (*μ*, day^−1^); (**B**) cellular biological silicon content (fmol cell^−1^); (**C**) cellular chlorophyll *a* content (fg cell^−1^); (**D**) maximum photochemical quantum yield of PSII (*F*_*V*_/*F*_*M*_); (**E**) net photosynthetic O_2_ evolution rate (*P*_net_, fmol O_2_ cell^−1^ h^−1^); and (**F**) dark respiration rate (*R*_dark_, fmol O_2_ cell^−1^ h^−1^). The column shows the mean values and standard deviation (mean ± SD, *n* = 3). One-way ANOVA was used for statistical testing in panel A, and panels B–F were tested using a two-way ANOVA. *P < 0.1; **P < 0.01; and ****P < 0.0001; ns, not significant.

Si enrichment strongly enhanced the cell growth rate (*μ*) of *Synechococcus* sp. PCC 7002 during the 5-day cultivation (*F*_4,10_ = 16.23, *P* < 0.01) (Fig. 1A). The growth rate of cells grown under Si—0 μM was 0.28 ± 0.010 day^−1^ and increased with increasing Si concentration, accompanied by a maximal increase of 37% under Si—120 μM. We then measured cell component and physiological changes arising due to Si enrichment on days 1 and 5. Biological Si content under Si—0 μM was 0.16 ± 0.012 fmol cell^−1^ on day 1 and 0.15 ± 0.019 fmol cell^−1^ on day 5 (Fig. 1B). The bSi under Si—200 μM was 7.12 ± 0.24 fmol cell^−1^on days 1 and 3.33 ± 0.25 fmol cell^−1^ on day 5. Chlrophyll *a* content (Chl *a*) under Si—0 μM was 40.85 ± 1.12 fg cell^−1^ on day 1 and 36.43 ± 0.66 fg cell^−1^ on day 5, being increased by 42% and 12% under Si-200 μM on days 1 and 5, respectively (Fig. 1C). The maximum photochemical quantum yield (*F*_*V*_/*F*_*M*_) of PSII under Si—0 μM was 0.38 ± 0.017 on day 1 and 0.29 ± 0.008 on day 5 (Fig. 1D) and showed no significant trend with Si concentration (*F*_4,5_ = 0.18, *P* = 0.94), although positive effects of Si enrichment on the RLC-derived (Fig. S1A and B) maximum relative electron transport rate (rETR_max_) and saturation light (*E*_*K*_) occurred on both days 1 and 5 (Table 1). The net photosynthetic O_2_ evolution rate (*P*_net_) under Si—0 μM was 6.02 ± 0.16 fmol O_2_ cell^−1^ h^−1^ on day 1 and 4.49 ± 0.16 fmol O_2_ cell^−1^ h^−1^ on day 5, being increased by 86% and 11% under Si—200 μM on days 1 and 5, respectively (Fig. 1E). The dark respiration rate (*R*_dark_) (Fig. 1F) and the photosynthetic RLC-derived (Fig. S1C and D) maximum photosynthetic O_2_ evolution rate (*P*_max_) (Table 1) showed a similar trend of Si-enrichment effect as *P*_net_ on both days 1 and 5. Overall, these findings indicate that silicon plays a crucial role in enhancing the growth and photosynthetic efficiency of *Synechococcus* sp. PCC 7002, highlighting its potential importance in natural aquatic ecosystems.

**TABLE 1:** The rapid light curve-derived light utilization efficiency (*α*), saturation irradiance (*E*_*K*_, μmol photons m^−2^ S^−1^), maximal relative electron transport rate (rETR_max_), and maximal photosynthetic O_2_ evolution rate (*P*_max_, fmol O_2_ cell^−1^ h^−1^) of PCC 7002 grown under 0–200 µM silicon enrichment on days 1 and 5 (mean ± SD: *n* = 3)^*a*^

		Si concentration (μM)				
		0	25	50	120	200
α	Day 1	0.19 ± 0.014a	0.18 ± 0.009c	0.17 ± 0.003c,d	0.18 ± 0.004c	0.18 ± 0.001e
	Day 5	0.16 ± 0.007b	0.17 ± 0.006d	0.16 ± 0.001b	0.16 ± 0.003b	0.15 ± 0.004f
rETR_max_	Day 1	103.41 ± 4.22a	110.35 ± 9.02b	121.12 ± 3.34d	125.86 ± 4.16c,d	131.37 ± 1.84c
	Day 5	109.38 ± 4.79b	129.65 ± 2.42c	116.85 ± 2.68b	130.73 ± 6.25c	126.38 ± 1.83e
E_K_ (μmol photons m^−2^ s^−1^)	Day 1	545.58 ± 54.33a	599.02 ± 20.54a	696.1 ± 9.36b	696.71 ± 23.46b	745.03 ± 11.19c
	Day 5	690.89 ± 10.06b	769.92 ± 33.56c	734.89 ± 15.87d	815.37 ± 31.4e	838.56 ± 17.18e
P_max_ (fmol O_2_ cell^−1^ h^−1^)	Day 1	7.36 ± 0.15a	9.43 ± 0.57c	11.12 ± 0.92e	13.010 ± 1.02f	13.25 ± 0.32f
	Day 5	5.30 ± 0.13b	5.79 ± 0.18d	5.89 ± 0.43d	6.16 ± 0.11g	6.77 ± 0.20h

^*a*^ Different letters near the values indicate significant differences (*P* < 0.05).

### Transcriptional response of *Synechococcus* sp. PCC 7002 to Si enrichment

To uncover the mechanisms behind these physiological responses to Si enrichment, we further analyzed the transcriptome profiles of cells grown under five Si concentrations on day 5. There was a total of 35.84 Gb clean data extracted from 15 samples. Differentially expressed genes (DEGs) were identified with the thresholds of log_2_ fold change < −1 or log_2_ fold change >1, *P* < 0.05. When exposed to Si—25 μM, there were 14 DEGs (3 upregulated and 11 downregulated). At Si—50 μM, the number of DEGs increased significantly to 367 (142 upregulated and 225 downregulated). Under Si—120 μM, 310 DEGs were identified (121 upregulated and 189 downregulated). The highest number of DEGs was found at Si—200 μM, with a total of 503 (205 upregulated and 198 downregulated) (Fig. S2). The KEGG enrichment analysis revealed significant pathways associated with the differentially expressed genes under Si—200 μM compared to Si—0 μM conditions (Fig. S3). The top 20 enriched pathways are shown, with key pathways including carotenoid biosynthesis, carbon fixation, the tricarboxylic acid (TCA) cycle, and glycolysis. These findings indicate that the DEGs are closely associated with physiological responses such as changes in photosynthetic efficiency and growth rates.

**Fig 2:**
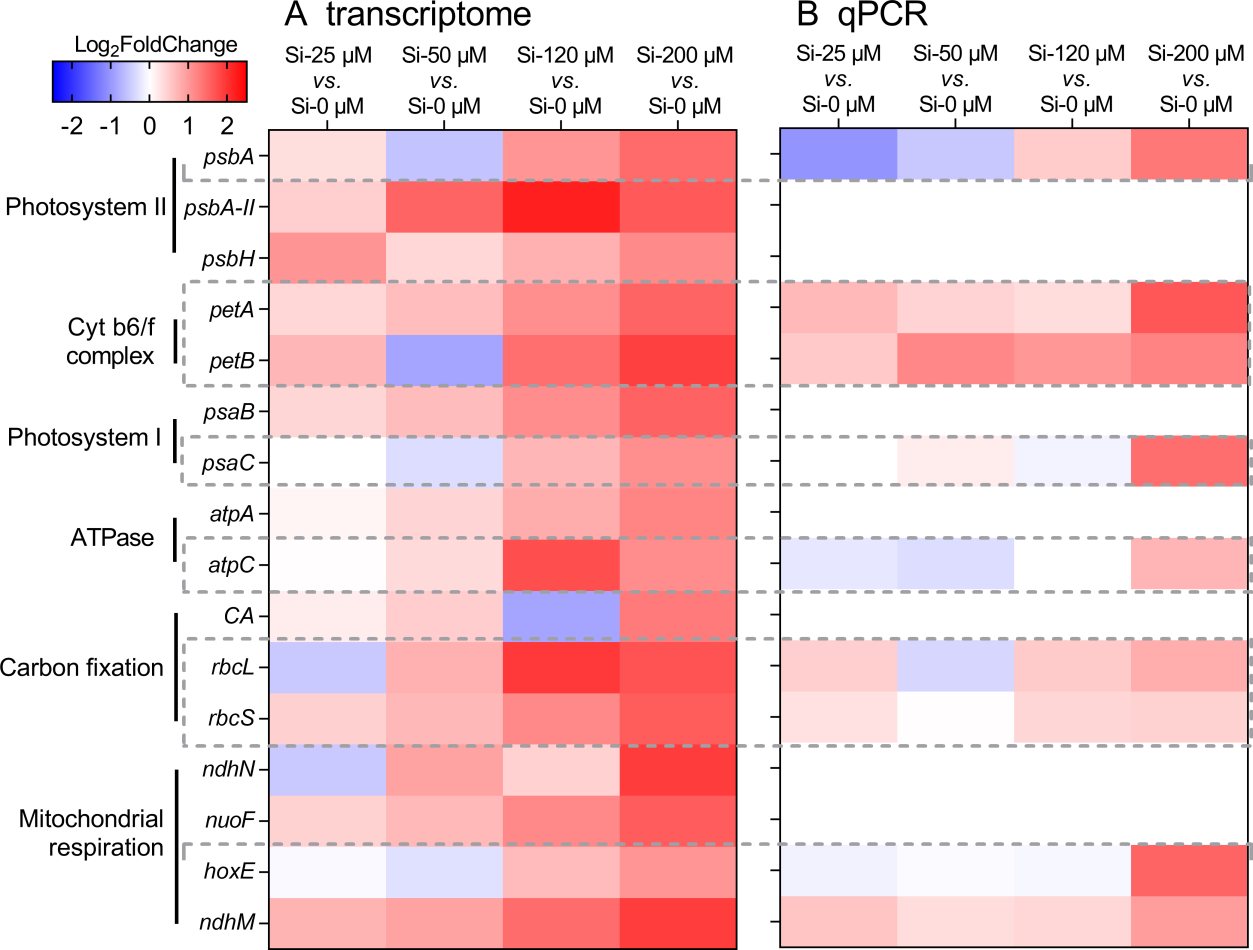
Relative expression of physiological genes of *Synechococcus* sp. PCC 7002 grown under silicon enrichment on day 5. (**A**) Transcriptomic regulation; (**B**) qPCR verification. *psbA,* photosystem II q(b) protein; *psbA-II,* photosystem II q(b) protein type II; *psbH,* photosystem II reaction center protein PsbH; *petA,* c-type cytochrome; *petB,* cytochrome b6-f complex subunit IV; *psaB,* photosystem I core protein PsaB; *psaC,* photosystem I iron-sulfur center protein PsaC; *atpA,* F0F1 ATP synthase subunit alpha; *atpC,* F-type H^+^-transporting ATPase subunit epsilon; *CA,* carbonic anhydrase; *rbcL,* ribulose bisphosphate carboxylase large subunit; *rbcS,* ribulose bisphosphate carboxylase small subunit; *ndhN,* NAD(P)H-quinone oxidoreductase subunit N; *nuoF,* NADH-quinone oxidoreductase subunit NuoF; *hoxE,* bidirectional hydrogenase complex protein HoxE; and *ndhM*, NADH-quinone oxidoreductase subunit M.

Based on the functional enrichment results, we focused on the expression of genes related to physiological functions at the Si—200 μM condition (Fig. 2A), such as PS II (*psbA* and *psbH*), Cyt b6/f complex (*petA* and *petB*), PS I (*psaB* and *psaC*), ATPase (*atpA* and *atpC*), CO_2_ concentrating mechanisms (*CA*), the large and small subunits of RubisCO (*rbcL* and *rbcS*), and the NADH-quinone oxidoreductase subunit (*ndhN*, *ndhM*, and *nuoF*) of mitochondrial respiration, and found that these important physiological function genes were variously upregulated by Si enrichment. Meanwhile, the downregulated genes were generally relevant to the secondary metabolism, e.g., purine-synthesizing genes *apt* and *purC* and pyrimidine-synthesizing genes *pyrE* and *pyrR* (Fig. S4). We then designed primers (Table S1) of nine genes for qPCR validation (Fig. 2B). All selected gene expressions increased with rising Si concentration. At 200 µM Si concentration, expressions of *psbA, petA, petB, psaC, atpC, rbcL, rbcS, hoxE, and ndhM* were upregulated by 1.314, 1.647, 1.206, 1.413, 0.717, 0.795, 0.435, 1.514, and 0.97 times, respectively. In summary, the expression of these nine genes in qPCR was consistent with the transcriptome. In brief, our analysis of transcriptome profiles reveals that silicon enrichment significantly impacts gene expression in *Synechococcus* sp. PCC 7002, with a substantial number of differentially expressed genes under high silicon concentrations. These DEGs are primarily involved in enhancing physiological functions related to photosynthesis and carbon metabolism, while genes associated with secondary metabolism are downregulated. These results highlight the crucial role of silicon in modulating gene expression to improve photosynthetic efficiency and growth rates in *Synechococcus* sp. PCC 7002.

### Function of transporters SySIT-L and SyLsi-L in Si uptake

**Fig 3:**
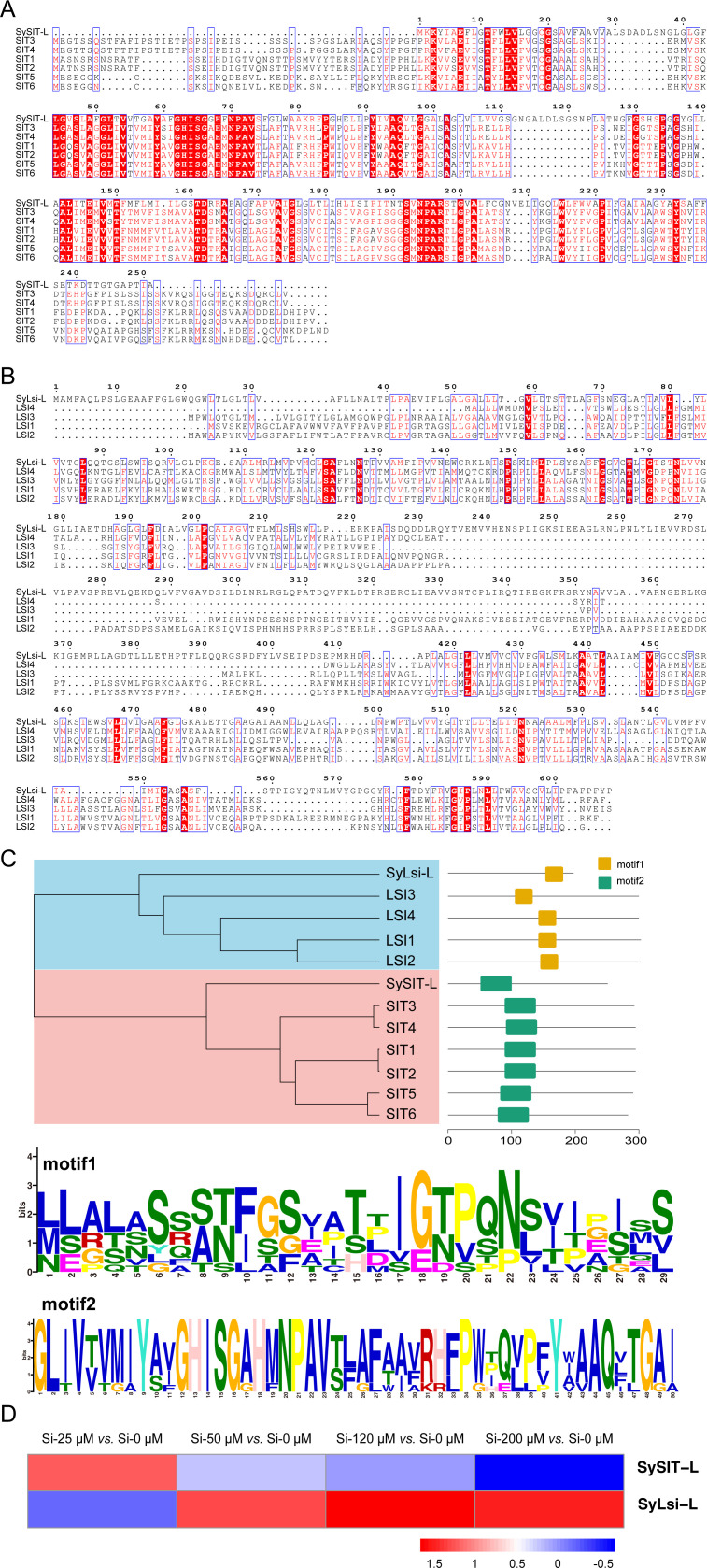
Sequence alignment of two putative Si transport proteins in *Synechococcus* sp. PCC 7002. (**A**) Sequence alignment of SySIT-L (Protein ID: WP_012308024.1). (**B**) Sequence alignment of SyLsi-L (Protein ID: WP_012306422.1). (**C**) Evolutionary analysis of SySIT-L and SyLsi-L. (**D**) Transcriptional levels of *SySIT-L* and *SyLsi-L*. SIT1: BAH24163.1; SIT2: ADM47602.1; SIT3: NP_001354442.1; SIT4: NP_001240190.1; SIT5: AKP80581.1; SIT6: NP_001274283.1; Lsi1: XP_001772790.1; Lsi2: XP_002984369.1; Lsi3: AFY62365.1; Lsi4: XP_013906816.1; and Lsi5: XP_007508130.1.

To explore how Si enters the cells, we identified potential Si transporters in *Synechococcus* sp. PCC 7002. The identification was performed by HMMsearch (hidden Markov model search), and two proteins were found to probably be involved in silicon transport (SySIT-L and SyLsi-L). SySIT-L (WP_012308024.1) was similar to the silicon transporter protein GmNIP2-2 (SIT4) in soybean *Glycine max* (Fig. 3A) with an identity of 33.87%, which belonged to the aquaporin-like family (Fig. 3C). SyLsi-L (WP_012306422.1) showed a high similarity with the Lsi2-type silicon transporter protein SELMODRAFT_180959 (Lsi2) from *Selaginella moellendorffii* with an identity of 31.48% (Fig. 3B). The transcriptome data indicated that SySIT-L was induced at a Si concentration of 25 µM, but its expression decreased with further increases in silicon concentration. In contrast, the expression of SyLsi-L increased with rising silicon concentration (Fig. 3D). These results indicate that SySIT-L and SyLsi-L work synergistically in silicon transport, with SySIT-L playing a major role at low concentrations, and SyLsi-L taking over as the concentration increases. Based on the above, SySIT-L and SyLsi-L are likely involved in the Si uptake of *Synechococcus* sp. PCC 7002.

**Fig 4:**
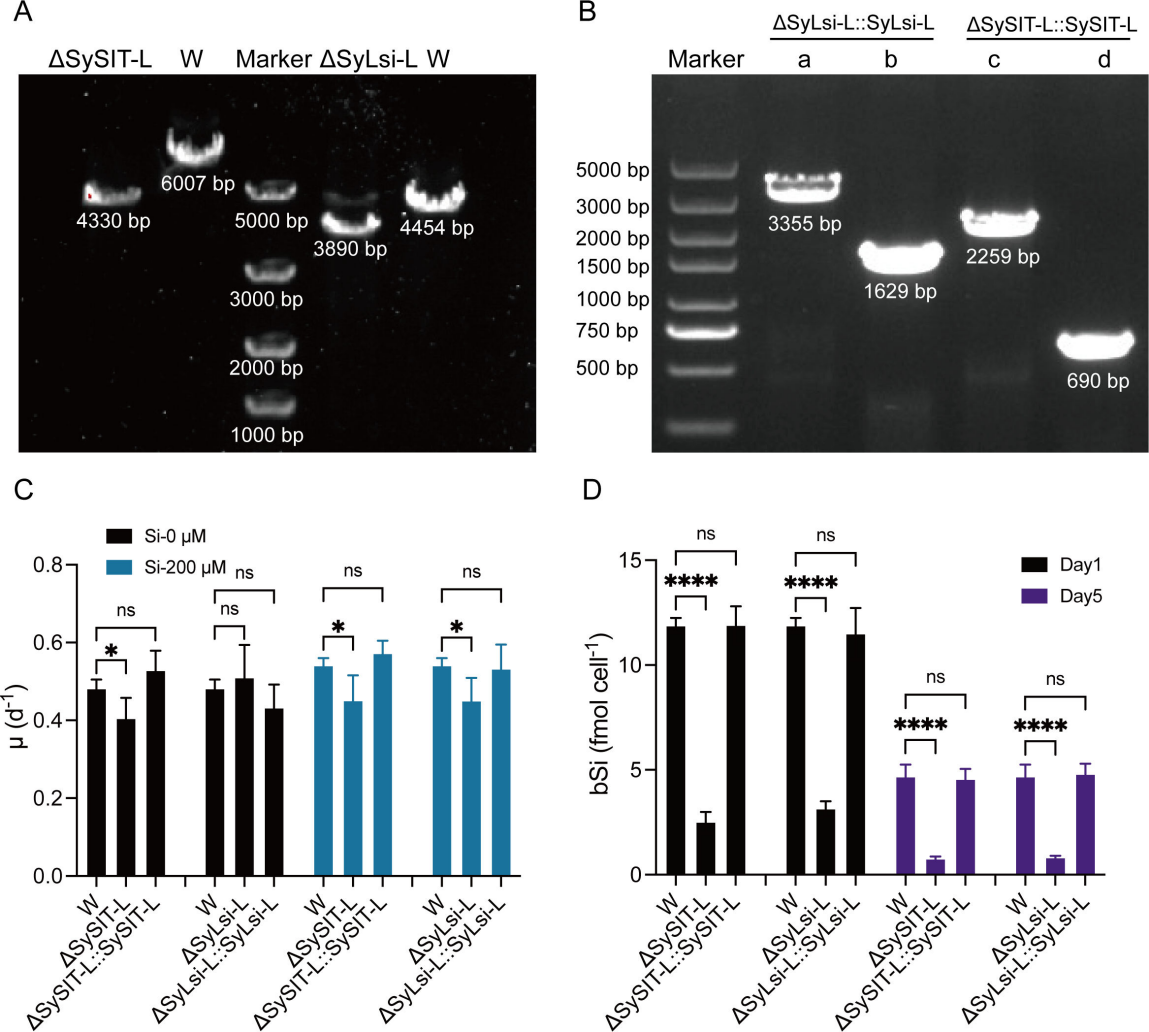
The effect of SySIT-L and SyLsi-L on the growth rate and cellular biological silicon content of *Synechococcus* sp. PCC 7002. (**A**) Gene deletion of SySIT-L and SyLsi-L verified by agarose gel electrophoresis. (**B**) Complemented strain verification. Primers NS1-F/R were designed for validation upstream and downstream of the NS1 site (**A and C**); primers Lsi-F/R and SIT-F/R were designed for validation within the genes SyLsi-L and SySIT-L (**B and D**). (C) Specific growth rate (*μ*, day^−1^) of *Synechococcus* sp. PCC 7002 and the knockouts and the complemented strains grown under 0 and/or 200 µM silicon enrichment. (**D**) Cellular biological silicon content (fmol cell^−1^) of *Synechococcus* sp. PCC 7002 and the knockouts and the complemented strains on days 1 and 5. Column shows the mean values and standard deviation (mean ± SD, *n* = 3), and *P < 0.1; **P < 0.01; and ****P < 0.0001; ns, not significant (two-way ANOVA).

The functions of SySIT-L and SyLsi-L were further verified by gene deletion and complementation. The knockout strains PCC7002Δ*SySIT-L* and PCC7002Δ*SyLsi-L* were obtained by homologous recombination gene knockout (Fig. 4A), and the complemented strains were constructed by inserting the *SySIT-L* and *SyLsi-L* genes into the NS1 site of the knockout strains (Fig. 4; S5B). Then, we cultured the wild-type, knockout strains, and complemented strains under Si—0 μM and Si—200 μM for 5 days. The growth rate of *Synechococcus* sp. PCC 7002 under Si—0 μM was 0.41 ± 0.009 day⁻¹ and increased by 10% under Si—200 μM (Fig. 4C). Compared to *Synechococcus* sp. PCC 7002, the growth rate of Δ*SySIT-L* and Δ*SyLsi-L* was reduced under both Si—0 μM and Si—200 μM conditions. After complementing the respective genes, the specific growth rates of the strains were restored to levels comparable to the wild type. On day 1, bSi content of *Synechococcus* sp. PCC 7002 was 11.87 ± 0.30 pmol cell^−1^ and was decreased by 79% and 74% in Δ*SySIT-L* and Δ*SyLsi-L*, respectively. The bSi content of *Synechococcus* sp. PCC 7002 on day 5 was 61% lower than that on day 1 and was further decreased by 84% and 82% under Δ*SySIT-L* and Δ*SyLsi-L*, respectively. After complementing the respective genes, the bSi content of the strains Δ*SySIT-L:: SySIT-L* and Δ*SyLsi-L:: SyLsi-L* was restored to levels comparable to the wild type on both days 1 and 5 (Fig. 4D). Similarly, Chl *a* content, Fv/Fm, *P*_net_, and *R*_dark_ were both significantly increased by Si enrichment between the control, especially on day 1 (Fig. S6A through D), as well as the RLC-derived parameters (Fig. S7A through D). Compared to the control, most of these physiological parameters under Δ*SySIT-L* and Δ*SyLsi-L* were significantly decreased on day 1 and trending to be insignificant on day 5. These findings demonstrate that both SySIT-L and SyLsi-L play significant roles in silicon uptake in *Synechococcus* sp. PCC 7002, underscoring their importance in the silicon-dependent physiological processes of the organism.

**Fig 5:**
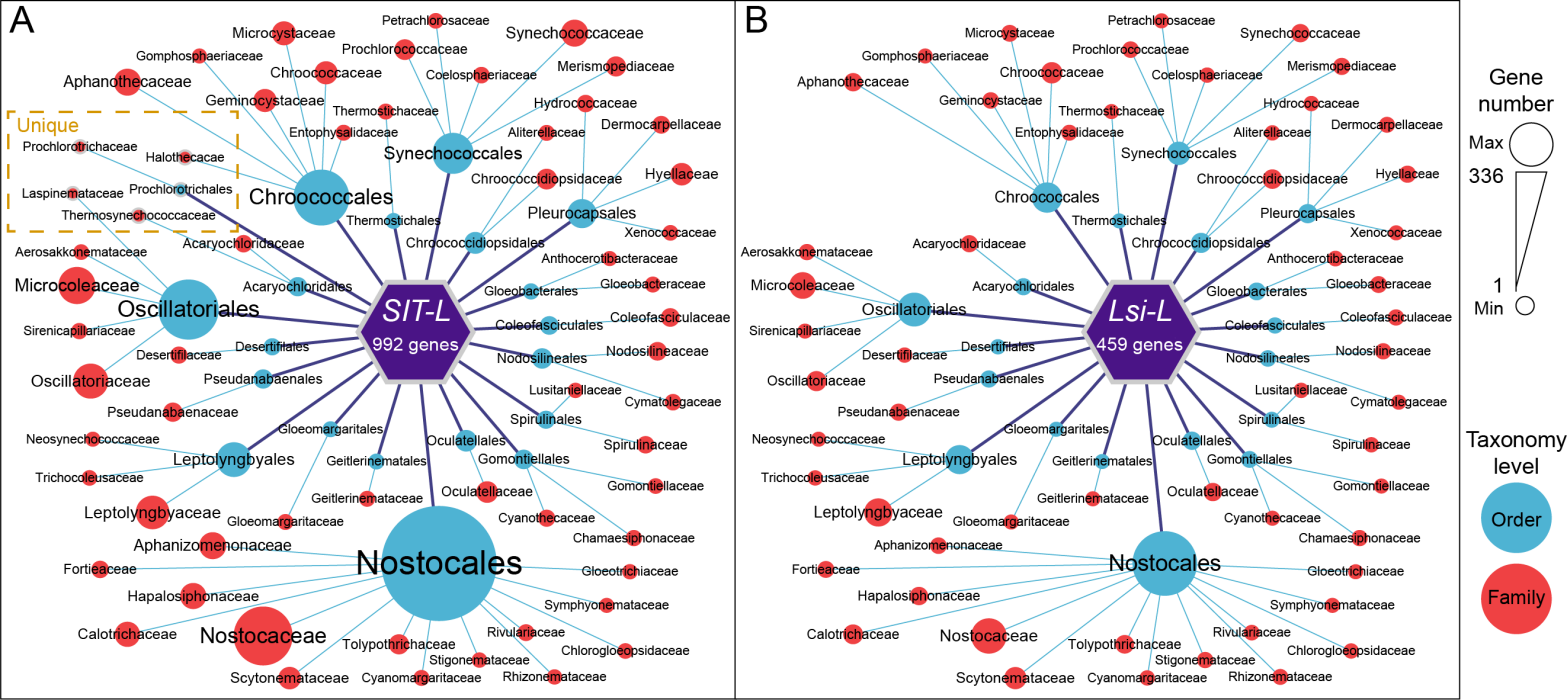
Distribution of SIT-L (**A**) and Lsi-L (**B**) in *Cyanobacteria*. Details are provided in Table S3.

### The distribution of SIT-L and Lsi-L in all sequenced cyanobacteria

We further analyzed the presence of SIT-L and Lsi-L in 469 sequenced cyanobacterial genomes (NCBI database, updated on 15 May 2023). Details are provided in Table S3. We showed the distribution of SIT-L and Lsi-L at the taxonomy levels of order and family (Fig. 5). There were 992 SIT-L distributed among 20 orders and 59 families (Fig. 5A). At the order level, the largest number was *Nostocales* (336), and the smallest was *Prochlorotrichales* (2). At the family level, *Nostocaceae* had the largest number (148), while four families, e.g., *Anthocerotibacteraceae* and *Neosynechococcaceae,* had only 1 gene. By contrast, the total number of Lsi-L decreased by 54% and was distributed among 19 orders and 55 families, with the highest numbers occurring in *Nostocales* and *Nostocaceae,* which decreased by 50% and 55%, respectively (Fig. 5B). In the genus *Synechococcus,* to which *Synechococcus* sp. PCC 7002 belongs, there were 36 SIT-L and Lsi-L, respectively. To sum up, our analysis of 469 cyanobacterial genomes reveals a widespread distribution of SIT-L and Lsi-L, with a notable prevalence in specific orders and families. The genus *Synechococcus*, which includes *Synechococcus* sp. PCC 7002, possesses a considerable number of these potential silicon transporters, suggesting their potential importance in silicon metabolism across diverse cyanobacterial taxa.

## DISCUSSION

**Fig 6:**
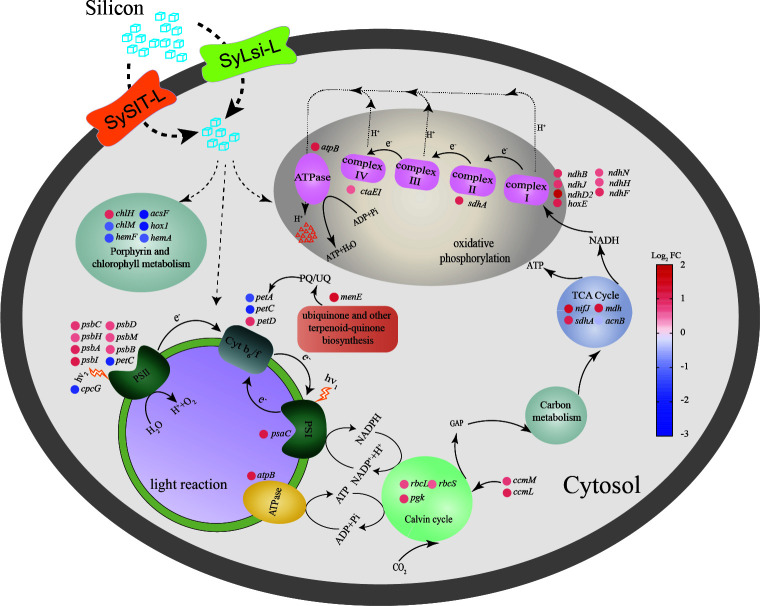
Silicon uptake promotes the growth and photosynthesis in *Synechococcus* sp. PCC 7002. Si enters the cell via transporters SySIT-L and SyLsi-L and promotes cell growth and photosynthesis by regulating the expression of key genes involved in photosynthesis and respiration.

Previous studies have observed the phenomenon of Si accumulation in picophytoplankton, including *Synechococcus* (6, 10, 11), but the insights into the mechanisms of how Si enters cells and how it affects cell metabolism are still limited (7, 13). Our data indicate that Si enhances the photosynthetic and respiratory processes of *Synechococcus* sp. PCC 7002, with Si uptake facilitated by two transporters, SySIT-L and SyLsi-L (Fig. 6). Previous research has also identified cyanobacterial EPS and extracellular aggregates that appear to speed up the precipitation of Si as magnesium silicates around cells (24, 25). These findings suggest that Si may enhance marine carbon cycling by improving *Synechococcus* photosynthesis and other associated metabolic pathways, as demonstrated in our study. Overall, our experiments indicate that Si accumulation in *Synechococcus* plays a crucial role in marine Si and C cycles, shedding new light on their close interconnection.

Throughout the microbial community in the euphotic zone, *Synechococcus* is an essential autotrophic microorganism that plays a critical role in primary productivity in the oceans (26, 27). Marine picoplankton is estimated to contribute about 4 × 10^12^ mol Si (28), with the bSi content of picoplankton in local marine areas being as high as ~5 fmol cell^−1^ (10), although this is well below the level of oceanic Si concentration in μmol L^−1^ (29). Our results support those investigations and further reveal the reduction of accumulated Si with the increase of cell growth (Fig. 1A and B). We believe this phenomenon was attributed to rapid cell division, resulting in reduced Si allocation per cell, aligning with the growth dilution principle of Brzezinski et al. (13). On the other hand, Si is not the essential growth element for *Synechococcus* in biochemistry but can be deposited on extracellular and/or intracellular polymeric substance (30). The carbon use efficiency (Fig. S8) calculated by photosynthetic rate and respiratory rate showed no significant difference between five Si treatments (*F*_4,5_ = 0.26, *P* = 0.89), indicating that the additional energy from photosynthesis (Fig. 1E and Table 1) with Si enrichment is consumed by the increased cell activities (Fig. 1F). Therefore, we hypothesized that the Si entering the cell caused a positive stress on cellular metabolism.

The results of our study on photosynthetic physiology revealed that the accumulation of Si positively impacted the photosystem activity of *Synechococcus* sp. PCC 7002 (Fig. 1E and Table 1). This is consistent with previous findings in diatoms that net primary productivity was significantly slower in Si-limited media than in Si-sufficient media (31, 32). It has been found that the absence of Si led to a reduction in seven essential proteins within the PS II complex, resulting in a decrease in the Cyt b6/f complex and PS I, ultimately leading to decreased overall photosystem activity (32). An intriguing observation warrants further discussion: the impact of Si on the photosynthesis rate was more prominent on day 1 than on day 5. We propose two possible explanations for this observation. First, the higher Si content per cell on day 1 may have contributed to a more significant increase in the photosynthetic oxygen release rate. Second, Si is an external factor that has the most significant impact on algae initially, but over time, the algae adapt and regulate themselves to counteract the effect of Si.

The genes *psbA*, *psbB*, *psbC*, and *psbD* are responsible for assembling the core subunits of PSII, with *psbA* encoding the D1 protein that plays a vital role in the water-splitting and oxygen-evolving processes, while *psbB* and *psbC* encode proteins that contribute to chloroplast light harvesting (33, 34). The upregulation of the *psbA* gene enhances PS II and improves the electron transfer process by connecting to secondary electron acceptors such as Phe and Q_B_ (35). Additionally, *psbH* encodes peripheral proteins of PS II. At the same time, *psbI* was closely associated with PSII assembly in higher plants (34, 36), providing further evidence that intracellular Si stimulation enhanced PS II in our results. The exact mechanisms by which Si regulated the photosynthesis-related genes remain unclear. However, studies on diatoms provide some insights. Si can enhance photosynthesis by adjusting the pH value and increasing CO_2_ levels in diatom (37). The nano-structured silica shells in diatoms can also improve light collection efficiency, thereby strengthening the primary photochemical reactions of PSII and accelerating the electron transfer rate (38). Additionally, Si may also impact signal transduction components related to photosynthesis in diatom (39). Moreover, it was observed that the gene *petD* was upregulated, encoding the Cyt b6/f complex. This complex facilitates electron transfer from PS II to PS I, proton transfer, and NADPH production for ATP synthesis and CO_2_ fixation (40–42). This discovery supports the previous finding that Si limitation significantly impacts diatom electron transfer and NADPH production (32). The upregulated expression of *psaB*, a gene that encodes P700 chlorophyll, an apolipoprotein, and *psaC*, a gene that encodes its subunit, improves the ability of PS I to support primary photochemical processes (43). Additionally, *ndhD2* has been demonstrated to facilitate cyclic electron transfer in PS I and contribute to generating the thylakoid proton gradient (44). Therefore, the upregulated expression of *atpB* and *ndhD2* suggests enhanced ATP synthesis processes due to intracellular Si.

Si enhances the respiratory rate of *Synechococcus* sp. PCC 7002 (Fig. 1F). Transcriptome analysis revealed that *nifJ* plays a crucial role in the tricarboxylic acid cycle, responsible for converting pyruvate to acetyl-CoA, which enters the TCA cycle, making the enzyme a key regulator of the cycle’s flux. Succinate dehydrogenase and malate dehydrogenase are also important enzymes in the TCA cycle, and the increased expression of genes encoding these enzymes, *sdhA* and *mdh*, suggests that Si enhances the TCA cycle. The reducing power generated by the TCA cycle is utilized in the electron transport chain to provide energy for ATP synthesis, thereby indirectly enhancing oxidative phosphorylation and electron transfer processes (45). Furthermore, aconitate hydratase (COA) mediates the conversion of citrate to isocitrate, ensuring the smooth progress of the TCA cycle. However, previous research has indicated that mutant strains with reduced COA enzyme expression lead to increased photosynthetic rates and enhanced carbon fixation (45). Oxidative electron transfer and phosphorylation are crucial for maintaining photosynthetic carbon assimilation, enhancing photosynthetic assimilation, and dissipating excess reducing power in chloroplasts to protect them from photoinhibition (46).

The accumulation of Si in the cells of *Synechococcus* is considered to be widespread (6). However, the mechanism of Si accumulation in *Synechococcus* is not yet clear. We verified the role of two Si transport proteins, SySIT-L and SyLsi-L, involved in Si uptake in *Synechococcus* (Fig. 4). We also investigated additional transporters in cyanobacteria similar to those in diatoms or higher plants. However, most of these genes are not regulated by Si concentration, so their functions require further experimental validation. SySIT-L and SyLsi-L are the first experimentally confirmed Si transporters discovered in cyanobacteria, although previous studies suggested the presence of sequences similar to the SIT sequence in *Synechococcus* (18). Here, we initially verified the Si transport functions of SySIT-L and SyLsi-L via gene knockout. However, much remains to be explored in the future, such as the specific cellular mechanisms and regulatory pathways involved in silicon uptake and accumulation by SySIT-L and SyLsi-L, as well as the ecological roles and advantages of silicon accumulation in different environmental contexts.

These two Si transporters belonged to two distinct families of Si transporters: SySIT-L is highly similar to the SIT family sequence found in diatoms (47), while SyLsi-L is highly similar to the Lsi family sequence found in plants (17). SIT and Lsi are two entirely different Si transporter families in eukaryotes, present in taxonomically isolated lineages. The absence of homology between them suggests that they evolved independently or through horizontal gene transfer (HGT). However, recent research has identified the SIT and Lsi2 genes in siliceous haptophytes (48), suggesting that these gene families are more widely distributed in eukaryotes. Here, we find that both of these protein families are also present in *Synechococcus* and are widely distributed in cyanobacteria (Fig. 5), indicating that they are also common in bacteria. Bacterial Si transporters were hypothesized to have originated from eukaryote-to-prokaryote HGT, although there is currently no clear evidence for an eukaryotic source (18). Future research could further explore the evolutionary origins of these transporters, potentially through more detailed phylogenetic analyses and comparative genomic studies. Additionally, investigating the functional diversity of these transporters across different cyanobacterial lineages may provide deeper insights into their roles in silicon metabolism and cellular adaptation.

Si plays a crucial role in biological systems, particularly in eukaryotes, where its most significant function is facilitating the formation of biomineralized cellular structures (18). In plants, Si enhances stress resistance, growth, and physiological regulation (17). In diatoms, it is vital for cell wall formation, photosynthesis, and environmental adaptation (39). Although cyanobacteria lack silicified cell walls, their intracellular silicon accumulation is also critical. Si accumulation strengthens their stress resistance, impacts their environmental interactions and ecological role in marine systems, and may influence the vertical transfer of biogenic silicon and carbon to the seafloor (49). In summary, the widespread distribution of Si transporters suggests that the utilization of Si may not be limited to extensively siliceous organisms and indicates that Si plays a broad and important role in the evolution of life and the maintenance of many biological processes.

## Data Availability

The transcriptomic data are deposited on NCBI (BioProject: PRJNA1034689), and further inquiries can be directed to the corresponding authors.

## SUPPLEMENTAL MATERIAL

Supplemental Figures and Tables mbio.01844-25-s0001.docx Figures S1-S8; Tables S1 and S2; Table S3 caption.

Table S3 mbio.01844-25-s0002.xlsx Distribution of two silicon transporter in all sequenced cyanobacteria.

ASM does not own the copyrights to Supplemental Material that may be linked to, or accessed through, an article. The authors have granted ASM a non-exclusive, world-wide license to publish the Supplemental Material files. Please contact the corresponding author directly for reuse.
