# Supplementary material for: Silicon uptake via the transporters SySIT-L and SyLsi-L enhances the growth and photosynthesis of Synechococcus sp. PCC 7002
Source: mBio. 2025 Aug 25;16(10):e01844-25. doi: 10.1128/mbio.01844-25 (PMC12506029; doi:10.1128/mbio.01844-25)
Supplement: Supplemental Figures and Tables — Figures S1-S8; Tables S1 and S2; Table S3 caption. [file mbio.01844-25-s0001.docx]

Silicon uptake via transporter SySIT-L and SyLsi-L enhances the growth and photosynthesis of *Synechococcus* sp. PCC 7002

Daixi Liu^a^, Bokun Chen^b^*, Yue Meng^b^, Yafei Wang^b^, Wei Zhao^b^, Hongli Ji^b^, Xue Yang^b^, Minghao Zhu^b^, Liwen Zheng^b,c^, Gang Li^d^, Jihua Liu^b,c,e^*

^a^State Key Laboratory of Discovery and Utilization of Functional Components in Traditional Chinese Medicine, Key Laboratory of Chemical Biology (Ministry of Education), Shandong Basic Science Research Center (Pharmacy), School of Pharmaceutical Sciences, Cheeloo College of Medicine, Shandong University, Jinan 250012, China

^b^Institute of Marine Science and Technology, Shandong University, Qingdao 266237, China

^c^Qingdao Key Laboratory of Ocean Carbon Sequestration and Negative Emission Technology, Shandong University, Qingdao 266237, China

^d^Daya Bay Marine Biology Research Station, South China Sea Institute of Oceanology, Chinese Academy of Sciences, Guangzhou 510530, China

^e^Global Ocean Negative Carbon Emissions (ONCE) Program Alliance

*Correspondence:

Jihua Liu: liujihua1982@foxmail.com;

Bokun Chen: chenbokunimst@163.com;

Correspondence ORCID: <https://orcid.org/0000-0001-6390-8364>

**Supplementary materials**

**Figure S1.** The rapid light curve (RLC) of rETR and gross photosynthetic O_2_ evolution rate (P_gross_, fmol O_2_ cell^-1^ h^-1^) of *Synechococcus* sp. PCC 7002 grown under 0 to 200 μM silicon enrichment at day 1 and day 5.

**Figure S2.** Volcano plots of DEGs under different silicon concentrations.

**Figure S3.** KEGG enrichment analysis of DEGs.

**Figure S4.** Relative expression of down-regulated genes of *Synechococcus* sp. PCC 7002 grown under silicon enrichment at day 5.

**Figure S5.** The plasmid map of the complemented strains.

**Figure S6.** The physiological changes of the wild type and knockout of *Synechococcus* sp. PCC 7002 grown under 200 μM silicon enrichment at day 1 and day 5.

**Figure S7.** The rapid light curve (RLC) of rETR and gross photosynthetic O_2_ evolution rate (P_gross_, fmol O_2_ cell^-1^ h^-1^) of the wild type and knockout of *Synechococcus* sp. PCC 7002 grown under 0 and/or 200 μM silicon enrichment at day 1 and day 5

**Figure S8.** The carbon use efficiency (CUE, %) of *Synechococcus* sp. PCC 7002 grown under 0 to 200 μM silicon enrichment at day 1 and day 5.

**Table S1.** Primers used in this study

**Table S2.** Strains and plasmids used in this study

**Table S3.** LSIs and SITs in cyanobacteria

**Figure S1.** The rapid light curve (RLC) of rETR and gross photosynthetic O_2_ evolution rate (P_gross_, fmol O_2_ cell^-1^ h^-1^) of *Synechococcus* sp. PCC 7002 grown under 0 to 200 μM silicon enrichment at day 1 and day 5. The statistical results in the figure are whether all the points can be represented by one curve (mean ± SD: n = 3).


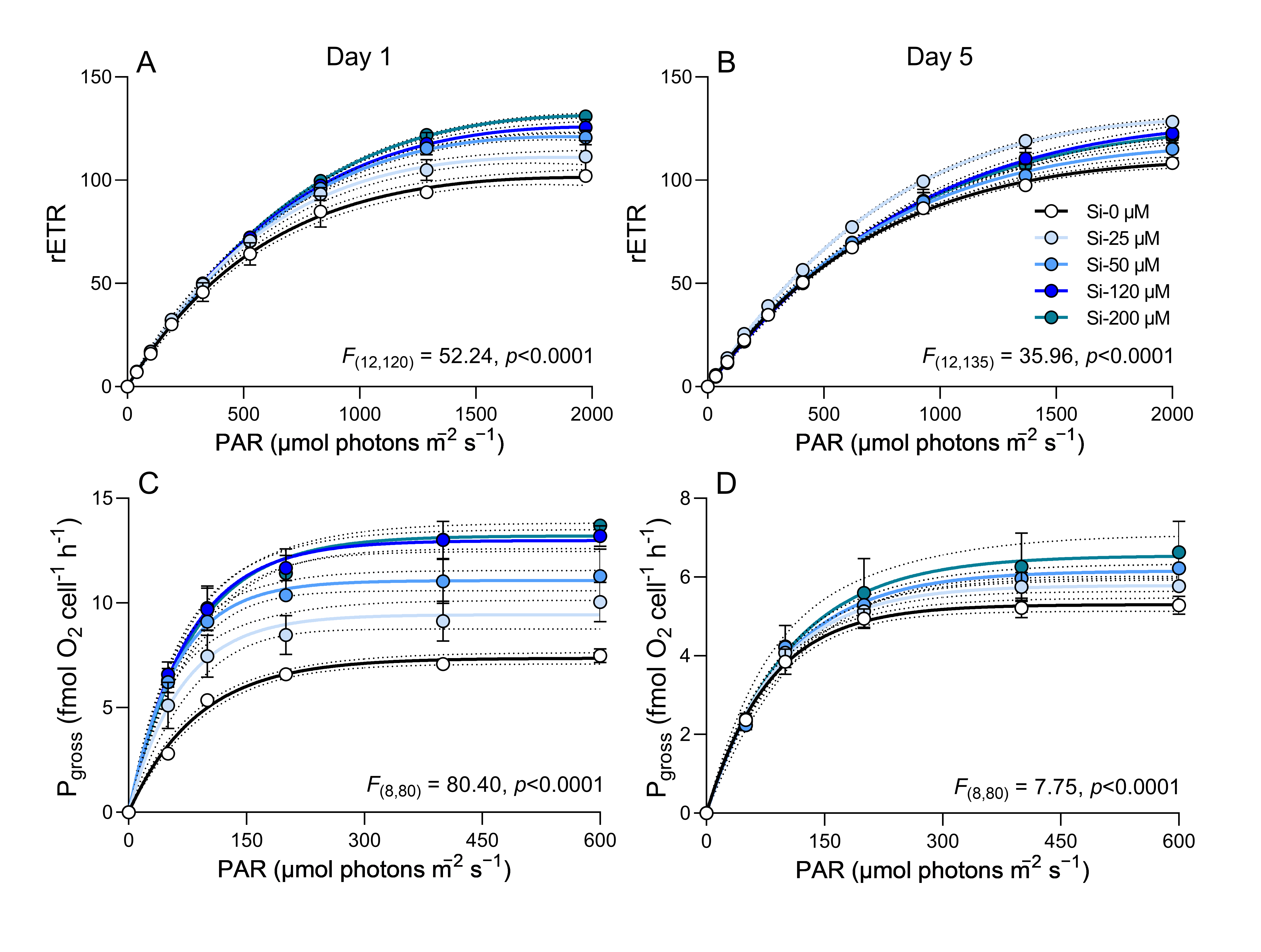


**Figure S2.** Volcano plots of DEGs under different silicon concentrations. A) Si-25 μM; B) Si-50 μM; C) Si-120 μM; D) Si-200 μM.


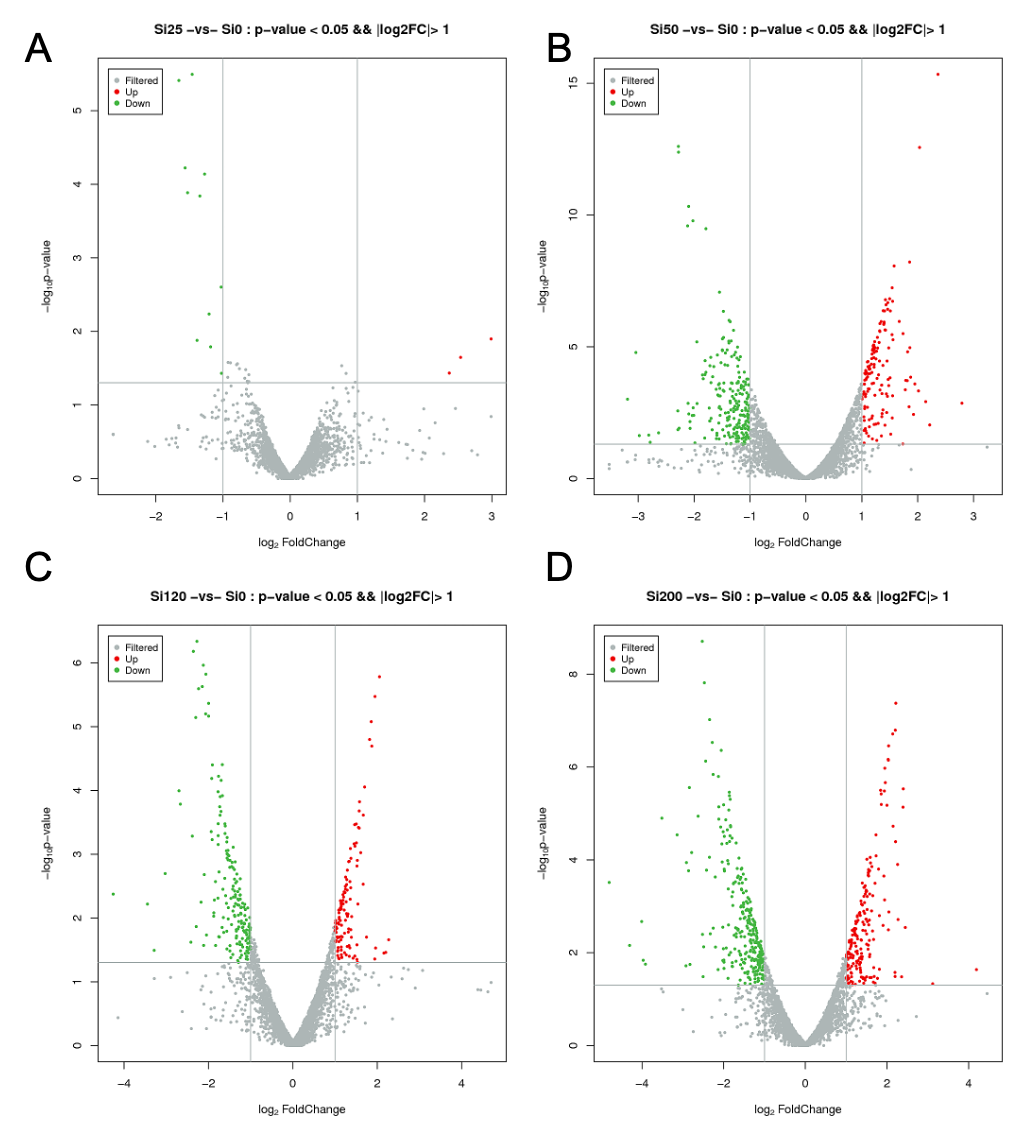


**Figure S3.** KEGG enrichment analysis of DEGs. The size of the dots represents the number of genes involved, while the color indicates the p-value, with red highlighting more significant results.


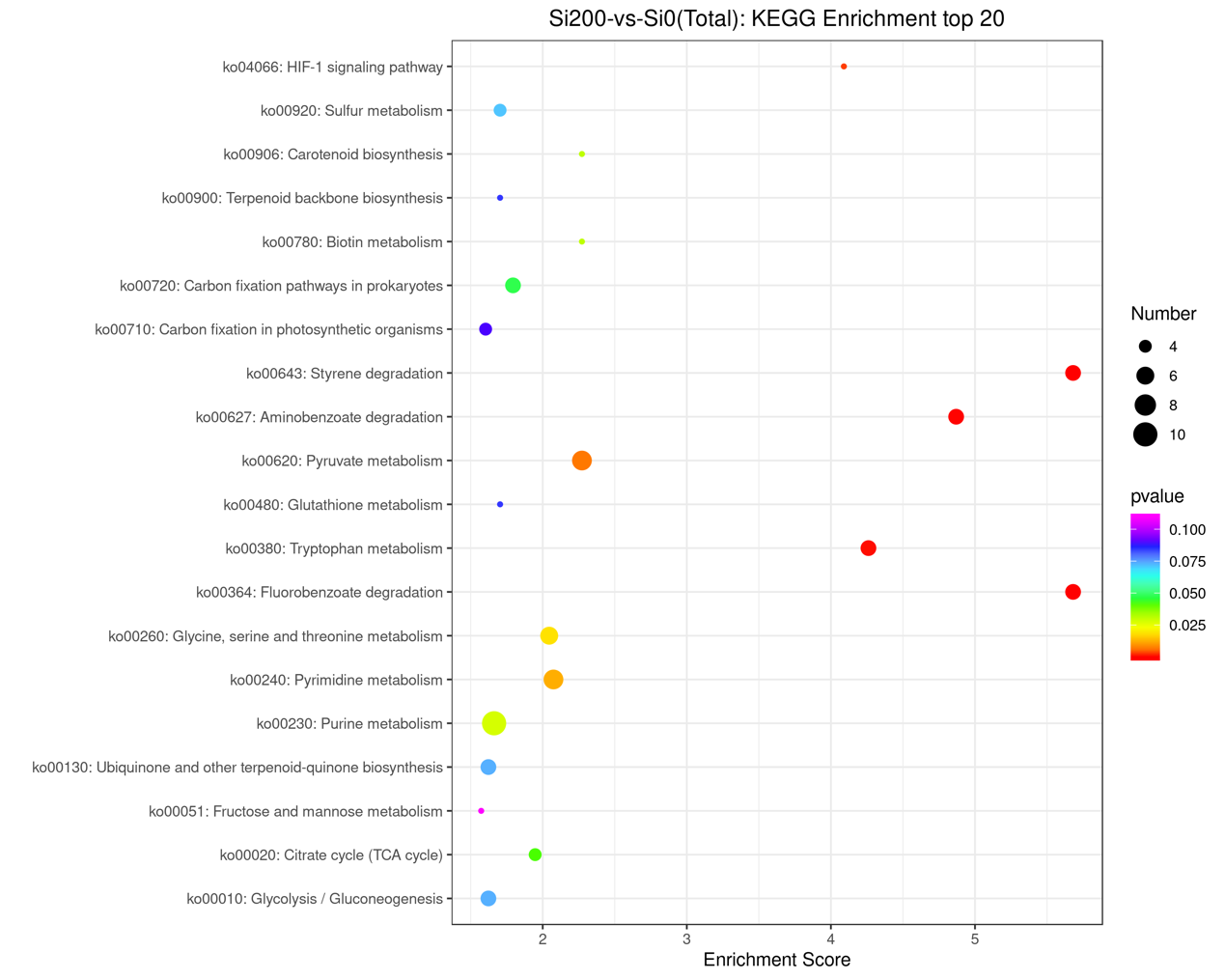


**Figure S4.** Relative expression of down-regulated genes of *Synechococcus* sp. PCC 7002 grown under silicon enrichment at day 5. *yhgE*: putative membrane protein; *livH*: high-affinity branched-chain amino acid ABC transporter; *ycgO*: putative Na+/H+ exchanger; *trpS*: tryptophanyl-tRNA synthetase; *pheS*: phenylalanyl-tRNA synthetase alpha chain; *fabZ*: beta-hydroxyacyl-(acyl-carrier-protein) dehydratase FabZ; *accB*: biotin carboxyl carrier protein; *accD*: acetyl-CoA carboxylase, carboxyl transferase, beta subunit; *GAPDH*: glyceraldehyde 3-phosphate dehydrogenase; *pdhAa*: Pyruvate dehydrogenase E1 component alpha subunit; *pdhB*: pyruvate dehydrogenase E1 component subunit beta; *tpiA*: triosephosphate isomerase; *nirB*: nitrite reductase (NADH) large subunit; *narB*: nitrate reductase; *yyaL*: nucleoside triphosphate pyrophosphohydrolase; *apt*: adenine phosphoribosylltransferase; *purC*: phosphoribosylaminoimidazole-succinocarboxamide synthase; *nadM*: bifunctional NMN adenylyltransferase/nudix hydrolase; *sat*: sulfate adenylyltransferase; *purT*: phosphoribosylglycinamide formyltransferase 2; *purH*: phosphoribosylaminoimidazolecarboxamide formyltransferase; *phnV*: putative 2-aminoethylphosphonate transport system permease; *pyrR*: Pyrimidine operon attenuation protein/uracil; *dcd*: Deoxycytidine triphosphate deaminase; *pyrE*: orotate phosphoribosyltransferase; *pyrC*: dihydroorotase; *carA*: carbamoyl-phosphate synthetase subunit A; *rps15*: 30S ribosomal protein S15; *rplU*: ribosomal protein L21; *rpsP*: small subunit ribosomal protein S16; *dxr*: 1-deoxy-D-xylulose-5-phosphate reductoisomerase; *idi*: isopentenyl-diphosphate Delta-isomerase; *desA*: fatty acid desaturase; *phoB*: two-component system, OmpR family, phosphate regulon response regulator; *glnB*: nitrogen regulatory protein P-II 1; *walR*: transcriptional regulator.


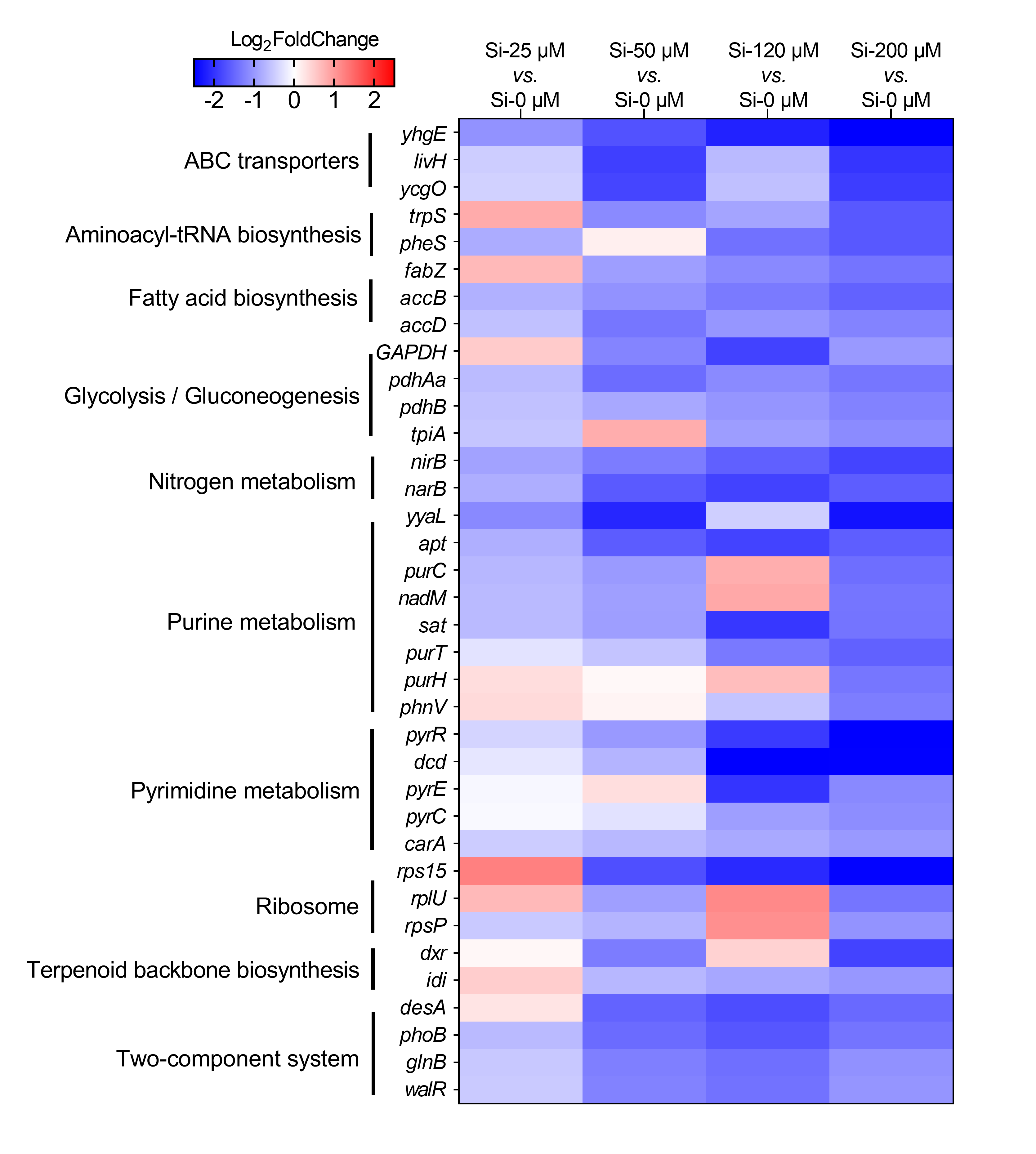


**Figure S5.** The plasmid map of the complemented strains. A) pJET-NS1-SyLsi-L; A) pJET-NS1-SySIT-L;


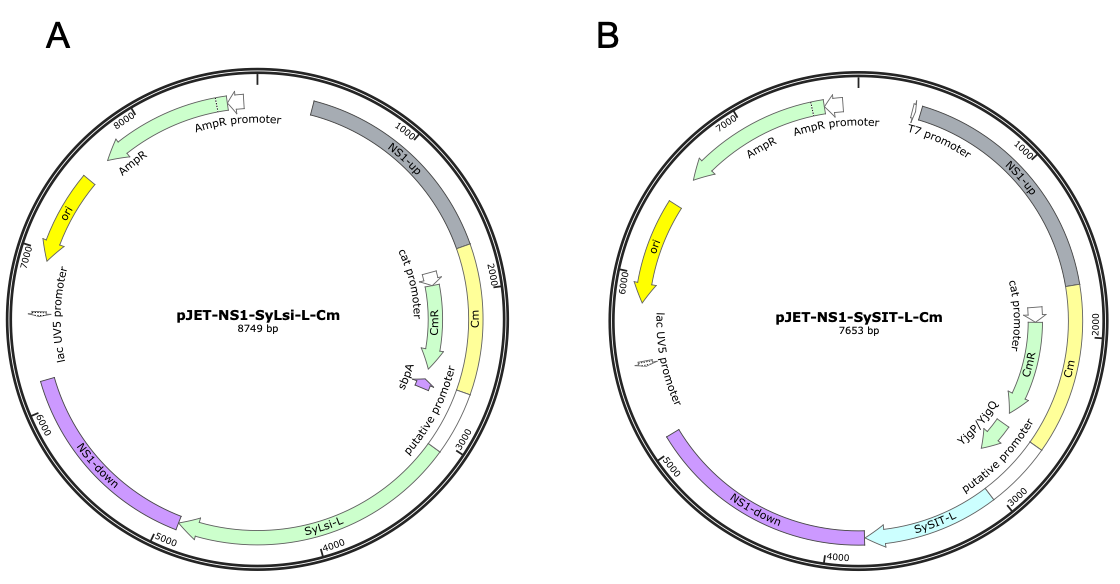


**Figure S6.** The physiological changes of the wild type and knockout of *Synechococcus* sp. PCC 7002 grown under 200 μM silicon enrichment at day 1 and day 5. Column show the mean values and standard deviation (mean ± SD, n=3), and d*, p < 0.1; **, p < 0.01; ****, p < 0.0001; ns, not significant (two-way ANOVA).


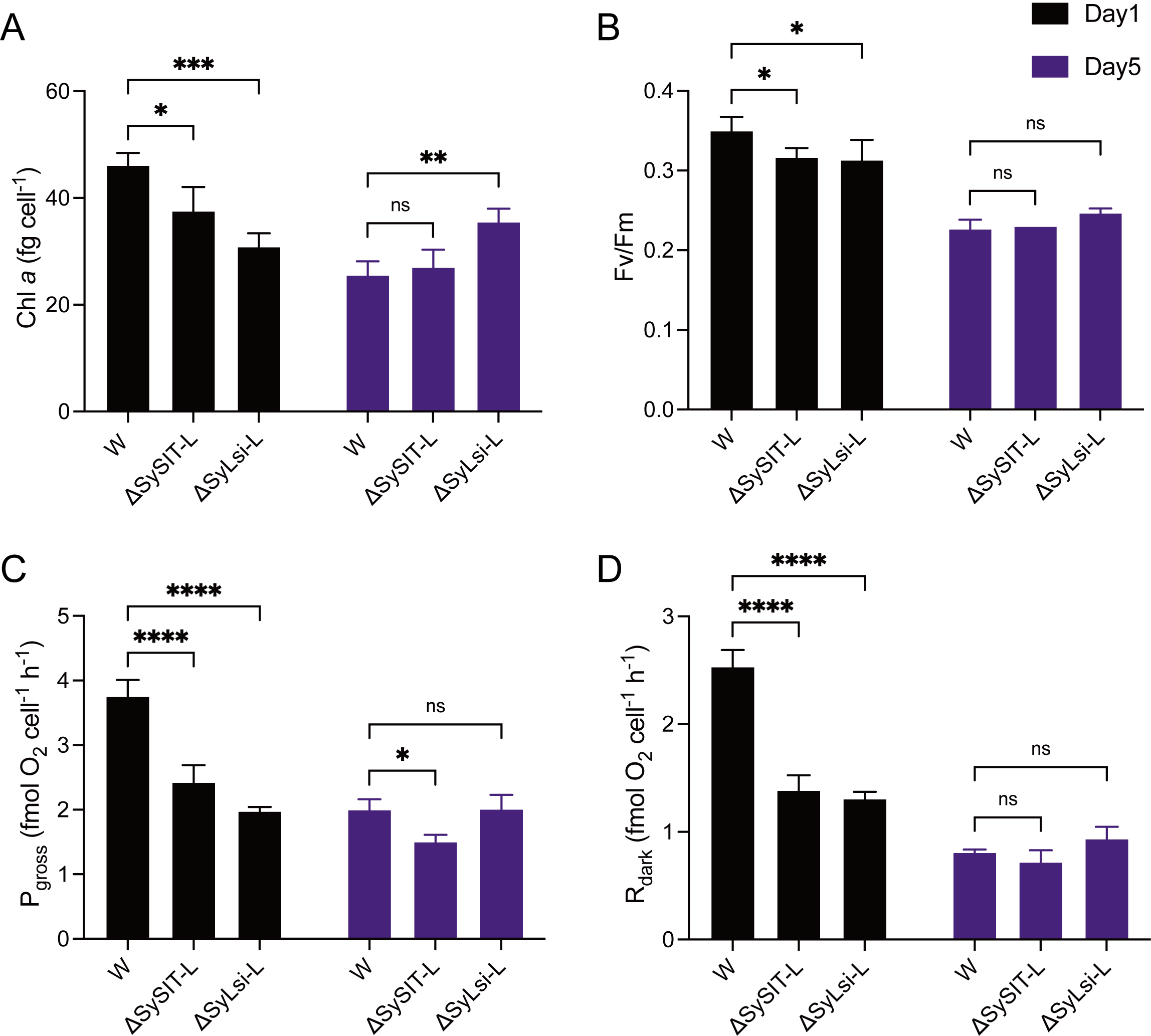


**Figure S7.** The rapid light curve (RLC) of rETR and gross photosynthetic O_2_ evolution rate (P_gross_, fmol O_2_ cell^-1^ h^-1^) of the wild type and knockout of *Synechococcus* sp. PCC 7002 grown under 0 and/or 200 μM silicon enrichment at day 1 and day 5. The statistical results in the figure are whether all the points can be represented by one curve (mean ± SD: n = 3).


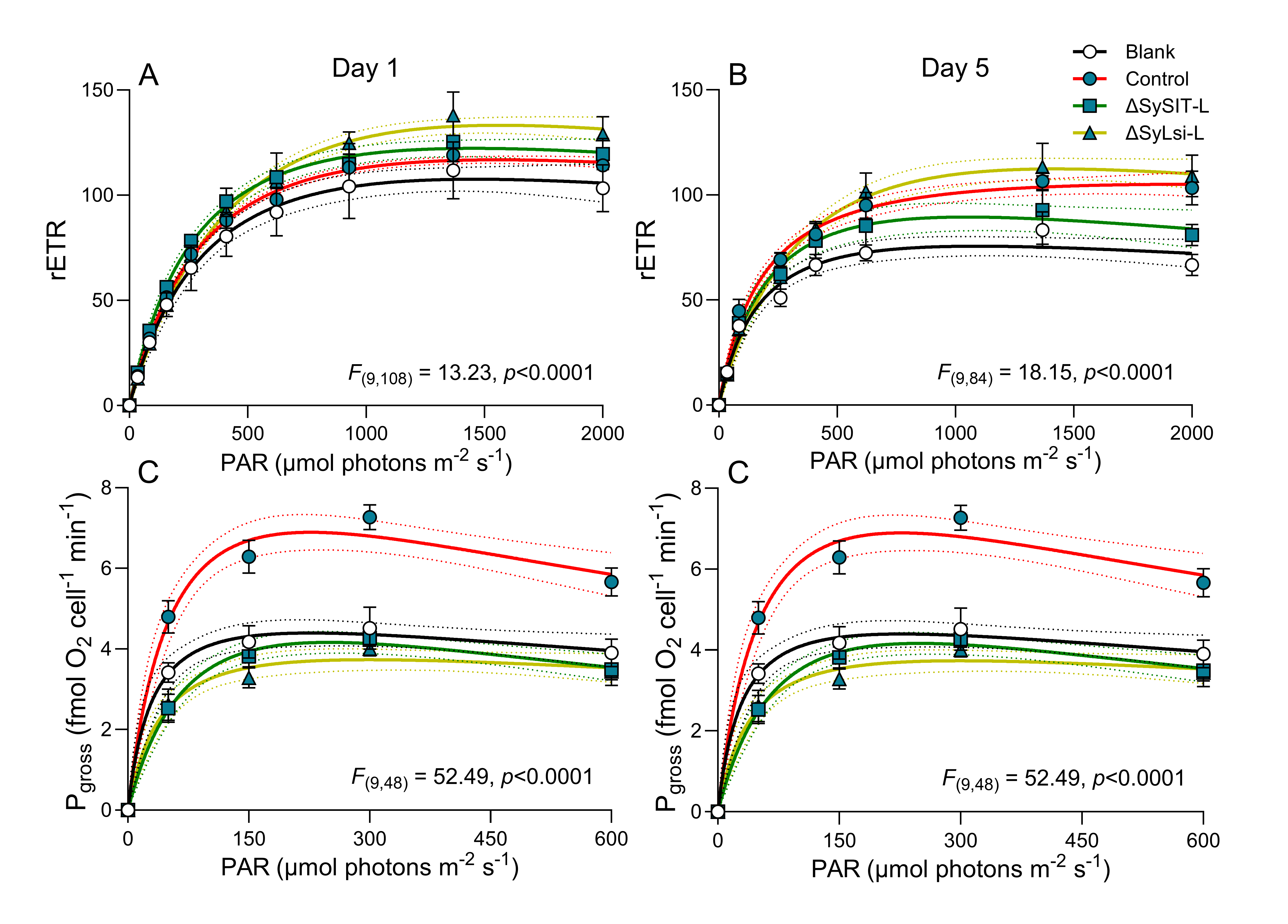


**Figure S8.** The carbon use efficiency (CUE, %) of *Synechococcus* sp. PCC 7002 grown under 0 to 200 μM silicon enrichment at day 1 and day 5. Column show the mean values and standard deviation (mean ± SD, n=3), and and d*, p < 0.1; **, p < 0.01; ****, p < 0.0001; ns, not significant (two-way ANOVA).


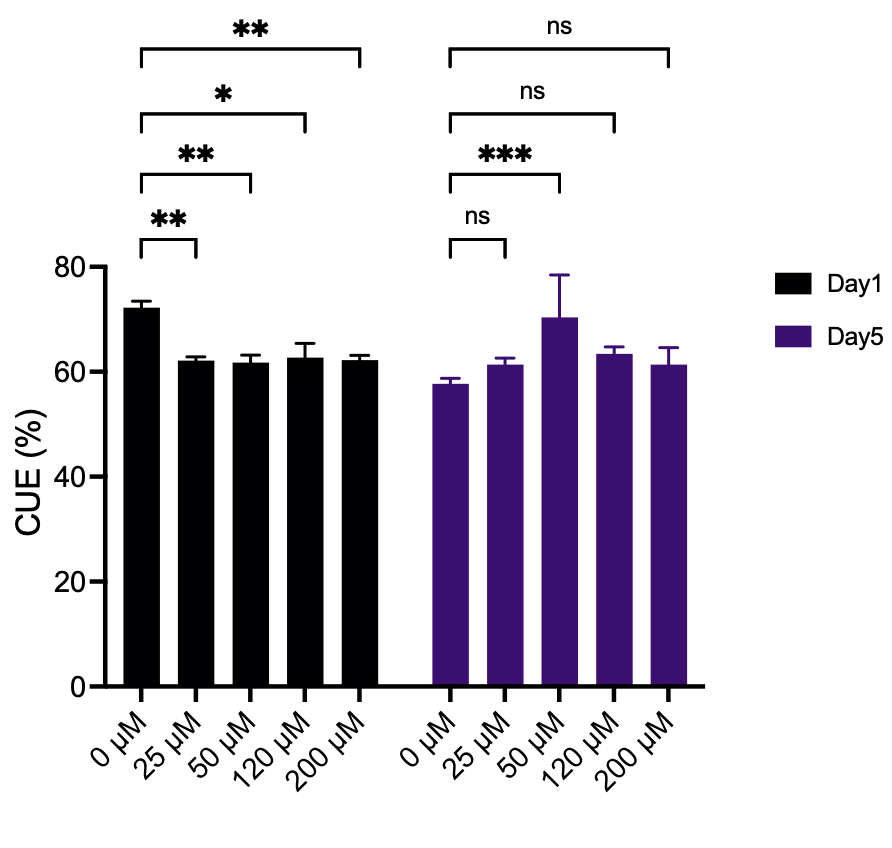


**Table S1.** Primers used in this study

| Name | | Sequence (5’→3’) |
| --- | --- | --- |
| *rbcS*-qPCR-F | ACAACACGGATGTAGCAGTC | |
| *rbcS*-qPCR-R | CGCCAAGTCCAGTACATGAT | |
| *rbcL*-qPCR-F | CGGAAGTGAATACCGTGGTT | |
| *rbcL*-qPCR-R | AAATCGGCACTCCCATCATC | |
| *psbA*-qPCR-F | CTTGGTGTGGCTGGTGTATT | |
| *psbA*-qPCR-R | ATCAAACGACCGAAGTAGCC | |
| *psaC*--qPCR -F | TCCTAGAGATGGTTCCTTGGG | |
| *psaC*--qPCR -R | TAGGCCAGACCCATACTACG | |
| *petA*--qPCR -F | TGTGATCATTGGGCCGATTT | |
| *petA*--qPCR -R | GCTTAGTTCACCAGTGGGAT | |
| *petB*--qPCR -F | GAAGTGAACTTCGGGTGGTT | |
| *petB*--qPCR -R | AGTTACACCGAAGGAAACGG | |
| *atpC*--qPCR -F | CATGGAATACGCCCTCGAAT | |
| *atpC*--qPCR -R | GCCAATTCACCTTCGGGTAA | |
| *hoxE*--qPCR -F | ATTTCCCACATGGGCTTTGT | |
| *hoxE* --qPCR -R | CATCGAGCATGAGGGTATGG | |
| *ndhM*--qPCR -F | TGCAAGCCCTGGGATTAAAG | |
| *ndhM*--qPCR -R | CAGCAAGGATCATGGAGACC | |
| *rnpA*--qPCR -F | GCCCCAACCAAAATTGGCATCAG | |
| *rnpA*--qPCR -R | TACCGCACAGCAATGACGATGTG | |
| *Kan-F* | GGCGATTAAGTTGGGTAACGCC | |
| *Kan-R* | AGGTTTCCCGACTGGAAAGCG | |
| *SySIT-L-del-1* | CGCTTTCCAGTCGGGAAACCTCGACATTAAAGGGCAGAATCGCG | |
| *SySIT-L-del-2* | TCGATTTTCCATGGCAGCTGAGCCAAAGAATTTTGGTTGTGCTTCG | |
| *SySIT-L-del-3* | GCCGCCAGATCTTCCGGATGGCATTTTTTGTTCGTAGAGGGGATAGCC | |
| *SySIT-L-del-4* | GGCGTTACCCAACTTAATCGCCGCTTTATAGCGAATTCCAAGCCC | |
| *SyLsi-L-del-1* | CGCTTTCCAGTCGGGAAACCTCCTGATATTTCCCCCATCGC | |
| *SyLsi-L-del-2* | GATTTTCCATGGCAGCTGAGCCTGGGCATTAGCAATTACCTG | |
| *SyLsi-L-del-3* | CGCCAGATCTTCCGGATGGCCAAGAGAAAAACGCCTCTGGC | |
| *SyLsi-L-del-4* | CGTTACCCAACTTAATCGCCCAATGAAGAGGCGCTTTTTTGTC | |
| NS1-F | TCCACTAAGCGATCGTGTAGAGG | |
| NS1-R | TCCATGAACCTTCTCACAAAGGAG | |
| Lsi-F | TGACCCTCGGTCTGACCCTG | |
| Lsi-R | TAGTTTGGTAGCCGATGGGAGTC | |
| SIT-F | TCTAGGGACATTCTGGTTGGTTCT | |
| SIT-R | TCGCTAAAGAAAGCACTATAGGCAT | |
| LSi-NS1-Cm-4 | GTAGTCTTTTCGCTTGCGGTTCTGCCATTCATCCGCTTATTAT | |
| LSi-NS1-Cm-5 | ATAAGCGGATGAATGGCAGAACCGCAAGCGAAAAGACTAC | |
| Lsi-NS1-Cm-6 | GAGGCAGATTGCGGCTTACTTTAGGGGTAAAAGGGAAAGGC | |
| Lsi-NS1-Cm-7 | CCTTTCCCTTTTACCCCTAAAGTAAGCCGCAATCTGCCTCA | |
| NS1-Cm-1 | CGCCAGATCTTCCGGATGGCTCATGTGGTGGAATTGCCAG | |
| NS1-Cm-2 | CGTCACAGGTATTTATTCGTTGCGATCAACGGACACAAAC | |
| NS1-Cm-3 | GTTTGTGTCCGTTGATCGCAACGAATAAATACCTGTGACGGAAGATC | |
| NS1-Cm-8 | GATTTTCCATGGCAGCTGAGACCATTGATGGGGTTCATCG | |
| SIT-NS1-Cm-4 | CCACACCCAGGGCGAGACTCTGCCATTCATCCGCTTATTAT | |
| SIT-NS1-Cm-5 | ATAAGCGGATGAATGGCAGAGTCTCGCCCTGGGTGTGG | |
| SIT-NS1-Cm-6 | GAGGCAGATTGCGGCTTACTCTAAGCGATCGTCGGCG | |
| SIT-NS1-Cm-7 | GGGCGCCGACGATCGCTTAGAGTAAGCCGCAATCTGCCTCA | |

**Table S2.** Strains and plasmids used in this study

| Strain or plasmid | Description/characteristic | Source/reference |
| --- | --- | --- |
| Strains |  |  |
| PCC7002 | Wilde type | This study |
| PCC7002ΔSySIT-L | PCC7002 with *SIT-L* deletion | This study |
| PCC7002Δ*SyLsi-L* | PCC7002 with *Lsi-L* deletion | This study |
| *E. coli* DH5α | Cloning strain | Novagen |
| Plasmids |  |  |
| pJET1.2-Blunt | Cloning vector | Thermo Fisher |
| pJET- *SySIT-L*-del | *SIT-L* deletion vector | This study |
| pJET-*SyLsi-L*-del | *Lsi-L* deletion vector | This study |
| pJET-NS1- *SySIT* -L | *SIT-L* complementation vector | This study |
| pJET-NS1-*SyLsi*-L | *SyLsi*-L complementation vector | This study |
